# Carbon Capture and Utilization Through Microalgae and Carbonic Anhydrases: Opportunities, Challenges and Future Directions

**DOI:** 10.3390/biom16101388

**Published:** 2026-09-23

**Authors:** Alfonso Ferrara, Paola Imbimbo, Rocco Acciaio, Vincenzo Massimiliano Vivenzio, Simona Maria Monti, Daria Maria Monti

**Affiliations:** 1 Department of Chemical Sciences, University of Naples Federico II, Via Cinthia 4, 80126 Naples, Italy; alfonso.ferrara@unina.it (A.F.); paola.imbimbo@unina.it (P.I.); rocco.acciaio@unina.it (R.A.); 2 Institute of Biostructures and Bioimaging, CNR, Via Pietro Castellino 111, 80131 Naples, Italy; vincenzomassimilianovivenzio@cnr.it

**Keywords:** bio-CCU, carbonic anhydrases, microalgae, carbon capture, enzyme immobilization

## Abstract

The continuous increase in carbon dioxide (CO_2_) has intensified the need for sustainable technologies to mitigate greenhouse gas emissions. Carbon Capture and Utilization (CCU) represents a promising strategy to convert captured CO_2_ into valuable products, contributing to climate change mitigation and the circular economy. This review provides an overview of the main carbon management pathways, including Carbon Capture and Storage (CCS), Carbon Capture, Utilization and Storage (CCUS), and CCU, with a focus on biological CCU (bio-CCU) based on microalgae and carbonic anhydrases (CAs). Particular attention is given to the role of CAs as highly efficient biocatalysts that accelerate CO_2_ hydration, enhancing inorganic carbon availability. Recent advances in enzyme immobilization technologies and support materials are examined, highlighting their potential to improve enzyme stability, reusability, and process performance. Furthermore, the integration of immobilized CAs with microalgal cultivation systems is explored as an emerging strategy in which CAs facilitate inorganic carbon availability and delivery, potentially enhancing its photosynthetic fixation by microalgae and biomass productivity. Building on previous studies on microalgal bio-CCU and CA-assisted carbon capture, this review provides an integrated perspective on CA immobilization strategies, support materials, and their potential implementation in microalgal bio-CCU systems, highlighting how enzyme immobilization can connect enzymatic CO_2_ conversion with microalgal carbon fixation and contribute to sustainable CO_2_ valorization and environmental remediation.

## 1. Introduction

More than 80% of global energy production comes from fossil fuel combustion, which is responsible for a substantial release of greenhouse gases (GHGs), such as CO_2_, CH_4_, N_2_O, and chlorofluorocarbons, with CO_2_ accounting for almost 70% of total emissions [1]. GHGs are essential for supporting life on Earth, as they maintain a stable climate, but an excess in GHG concentration leads to global warming and severe environmental consequences, including rising average temperatures, melting glaciers, sea-level rise, biodiversity loss, and the increasing frequency of extreme weather events [2]. Over the past decades, anthropogenic activities, such as rapid industrialization, urbanization, and energy demand, have significantly increased emissions, thus accelerating climate change and threatening natural ecosystems and human societies [1]. The persistent increase in atmospheric CO_2_ concentrations is one of the leading causes of the current climate crisis, with long-term impacts on water resources, food security, public health, and the global economy [2,3]. Consequently, reducing GHG emissions has become an urgent international priority. In response to these environmental concerns, several international agreements and climate policies have been established to limit global warming and to promote sustainable development. Among them, the Paris Agreement represents one of the major global commitments to fight climate change, aiming to limit the increase in global average temperature to below 2 °C compared to pre-industrial levels, while pursuing efforts to limit it to 1.5 °C [4]. To achieve these objectives, participating governments are working on the transition toward low-carbon and renewable energy systems [5].

Despite these international efforts, global emissions remain at critically high levels, thus highlighting the urgency for stronger environmental policies, technological innovation, and sustainable industrial practices [6,7]. In this context, setting up effective mitigation strategies could represent a crucial step to achieve climate neutrality and a more sustainable future. So far, approaches such as fuel switching, electrification, and energy efficiency improvements have been proven to be not sufficient to offset increasing fossil energy demand, especially in fast-growing regions [8]. As a result, research is focused on the development of complementary technologies capable of actively removing CO_2_ and, ideally, converting it into products with a high market value. Thus, innovative carbon capture technologies can represent a new challenge to decrease GHG levels, achieving net zero and, ultimately, net negative emissions. The deployment of these technologies, however, is strictly bound to the thermodynamic behavior of CO_2_, which can exist as a gas, liquid, or supercritical fluid, depending on pressures and temperatures. While these physical features allow different sequestration strategies, they also pose serious environmental and health risks due to their interactions with pipeline systems and geological storage formations, which can result in corrosion, leakage and material degradation [9].

Here, an updated review of scientific papers on different innovative carbon capture technologies is reported, with a special focus on biological processes involving microalgae. Microalgae are photoautotrophic microorganisms able to fix atmospheric CO_2_ and can therefore represent a biological tool for carbon capture [10]. Particular emphasis is given to three interrelated aspects: (i) enzymatic systems; (ii) materials used for enzyme immobilization; and (iii) microalgae. By integrating the available knowledge, this review aims to provide a critical perspective on the state-of-the-art of these methodologies, highlighting the potential of biological systems as environmentally friendly approaches for CO_2_ mitigation, while emphasizing that research in the field is still at an early stage.

## 2. Carbon Capture Technologies

Over the past decade, three main carbon capture strategies have been proposed: Carbon Capture and Storage (CCS), Carbon Capture Utilization and Storage (CCUS), and Carbon Capture and Utilization (CCU). Although they rely on common basic principles, they differ in the fate of captured CO_2_. In CCS, CO_2_ is captured, transported, and safely stored in suitable geological sites to meet emissions targets [11]. Taking advantage of well-established injection technologies, CO_2_ can be permanently stored in deep saline aquifers, depleted hydrocarbon reservoirs, or basalt formations, which offer enormous theoretical storage capacities estimated in thousands of Gigatons (Gts) [12,13]. A critical prerequisite for CO_2_ safe storage is a low-permeable or impermeable layer above the storage site to prevent potential leaks [14]. Saline aquifers are one of the most promising solutions due to the presence of water-bearing strata and their vast volumetric capacity. However, the elevated pressure may trigger fracturing and lead to CO_2_ leakage, posing risks to groundwater quality and thus necessitating stringent monitoring of the whole system and regulatory compliance [15]. On the other hand, depleted hydrocarbon reservoirs are estimated to store an amount of CO_2_ about 13 times higher than aquifers [15]. However, independent of the geological site, CCS deployment still faces major limitations, such as high operational costs (estimated at US$123–341/tCO_2_) [16], extensive infrastructure requirements, such as pipelines and storage hubs, and low public acceptance, particularly due to CO_2_ leaks [11,17]. Finally, CO_2_ can also be injected and stored into reactive basaltic rocks, where a carbonation reaction may occur due to the presence of carbon-fixing minerals. For this reason, basalt formations are considered a good long-term solution, as they allow permanent storage while minimizing the risk of leakage [14].

CCUS addresses CCS challenges by combining CO_2_ capture and long-term storage with its partial use as a carbon feedstock to produce fuels, chemicals, and construction materials. This technological framework relies on three consecutive stages: carbon capture, safe transportation and permanent storage [18]. By coupling CO_2_ mitigation with the generation of high-value products, the resulting revenue can support the transition toward more sustainable industrial systems.

However, despite its potential, CCUS exploitation is constrained by technological issues, social acceptance, the absence of defined regulatory frameworks and high investment costs. Many utilization pathways remain economically challenging, with costs often higher than CCS, contributing to the cancellation of 43% of announced projects [19]. In addition, CO_2_ utilization can increase overall energy consumption by 20–30% and may significantly increase water and land requirements, thereby introducing further environmental burdens [20]. Furthermore, possible CO_2_ leakage from storage sites, along with soil and ocean acidification, remains a risk with broader climate impact. These challenges are further intensified by complex supply chains, which increase infrastructure demands and logistical constraints, thus compromising the overall system efficiency.

Unlike the other strategies, CCU encompasses a variety of applications that rely on the use of captured CO_2_ from industrial sites or directly from the atmosphere, converting it through chemical, electrochemical, or biological processes into marketable products, without requiring transportation or geological storage [21]. CCU is primarily driven by economic value generation and emissions reduction, positioning captured CO_2_ as a strategic industrial feedstock rather than waste, thereby promoting the circular economy [22]. From a life-cycle assessment (LCA) perspective, CCU offers several distinct advantages that support its integration into industrial decarbonization strategies. First, by directly using captured CO_2_, CCU prevents its additional release into the atmosphere; second, by displacing carbon-intensive conventional products, it provides CO_2_-substitution credits; finally, by using renewable energy, it further reduces net emissions [22]. Consequently, CCU is strategically critical to reach EU net-zero objectives by 2050 and should be fully recognized and supported as a key tool to reduce emissions, especially in hard-to-abate sectors. Indeed, it has been estimated that CCU-derived products could reduce industrial GHG emissions by at least 20% [23]. In 2025, the global CCU market was estimated to be approximately USD 5 billion and is projected to grow at a compound annual growth rate (CAGR) of 25%, reaching USD 15 billion by 2033, driven by tightening regulatory frameworks, carbon pricing mechanisms, and increasing private investments. These data are even more encouraging when considering that global CCS was valued at approximately USD 4 billion in 2025 and is projected to reach USD 20 billion by 2034, growing at a CAGR of 18.0% from 2026 to 2034 [24]. Meanwhile, the global CCUS absorption market size was valued at about USD 6 billion in 2025, with a market projection up to USD 50 billion by 2034, exhibiting a CAGR of 25% during the forecast period [24]. Notably, the global CCU chemicals market was valued at USD 60 billion in 2024 and is expected to grow to about USD 500 billion in 2034, at a CAGR of 20.5% (according to the latest report published by Global Market Insights Inc., 6 May 2026). Thus, evidence underscores that CCU is not only a technically viable pathway but also an increasingly robust economic opportunity aligned with the EU 2050 net-zero ambitions.

While increasingly recognized as an effective decarbonization strategy, CCU still faces important technical and economic barriers, such as energy intensity [25]. Electrochemical and chemical conversions require substantial energy inputs, thus constraining scalability and economic competitiveness where low-cost, low-carbon electricity is not readily available [26,27]. Consequently, the search for less energy-intensive and more sustainable CO_2_ utilization routes has expanded beyond conventional physicochemical systems toward biological platforms, where carbon fixation is driven by microbial metabolism rather than by external energy-intensive catalysis.

## 3. Carbonic Anhydrases as Efficient Biocatalysts for Bio-CCU

Carbonic anhydrases (CAs, EC 4.2.1.1) are among the most promising biological tools for carbon capture and utilization due to their exceptional catalytic efficiency in CO_2_ conversion [28,29]. These enzymes, widely distributed in animals, plants, algae, and microorganisms, catalyze the reversible hydration of CO_2_ to bicarbonate and protons under mild conditions, according to the reaction:

CO_2_ + H_2_O ⇌ H^+^ + HCO_3_^−^

CAs are essential in several physiological processes, including respiration, photosynthesis, inorganic carbon transport, calcification, and acid–base regulation [30]. Their remarkably high turnover rates make them among the fastest known enzymes; certain isoforms, such as human carbonic anhydrase II (hCA II), can catalyze up to approximately 10^6^ CO_2_ molecules per second [31,32]. CA catalytic activity is usually mediated by a metal ion located in the active site, most commonly Zn^2+^, although other metal cofactors, such as Co^2+^, Mn^2+^, Fe^2+^, and Cd^2+^**^−^**, can occur depending on the enzyme family [33,34,35]. These metalloenzymes are currently classified into eight genetically distinct families, named α-, β-, γ-, δ-, ζ- η-, θ-, and ι-CAs, which are phylogenetically unrelated and differ in sequence, structure, metal cofactor preference, cellular localization, and organism distribution. For instance, α-CAs are widely represented in animals, plants, algae, fungi, protozoa, and bacteria [36,37,38], whereas β-CAs are common in plants, algae, cyanobacteria, and non-photosynthetic organisms [39,40,41]; γ-CAs have been identified in archaea, certain bacteria, and plants [41]. Other families show a more restricted distribution; for example, δ- and ζ-CAs are mainly reported in marine photosynthetic organisms [42,43], whereas η-, θ-, and ι-CAs have been identified more recently in selected protists, algae, diatoms, bacteria, and archaea [44,45,46]. This diversity reflects the multiple biological roles of CAs in inorganic carbon management, including respiration, pH regulation, photosynthesis, and Carbon-Concentrating Mechanisms (CCM). In CCM, CAs can accelerate the conversion of CO_2_ into bicarbonate and, in the presence of suitable cations, facilitate mineralization pathways leading to stable carbonate products such as CaCO_3_ [47]. Beyond carbonate precipitation, the enzymatically generated bicarbonate provides a soluble and bioavailable source of inorganic carbon for photosynthetic microorganisms such as microalgae and cyanobacteria. Therefore, these enzymes can support biological CCU routes by enhancing photosynthetic carbon assimilation, biomass accumulation [48], and the production of value-added bioproducts. hCA II (Figure 1A) is often considered a reference CA isoform due to its exceptionally high CO_2_ hydration activity, with kcat and kcat/K_m_ values of 1.4 × 10^6^ s^−1^ and 1.5 × 10^8^ M^−1^ s^−1^, respectively [49] and is thus frequently used as a model system for CCU. As schematically illustrated in Figure 1B, the catalytic mechanism of hCA II relies on the Zn^2+^ ion located in the active site, which is coordinated by three histidine residues and a water molecule. The metal-bound water is deprotonated to generate a Zn^2+^-bound hydroxide ion, which acts as a nucleophile and attacks CO_2,_ leading to the formation of a bicarbonate intermediate, which is subsequently released and replaced by a new water molecule, restoring the initial catalytic state. Through this cycle, hCA II efficiently catalyzes the reversible hydration of CO_2_ to bicarbonate and protons.

By accelerating CO_2_/HCO_3_^−^ interconversion through CA enzymatic activity, it is possible to mitigate kinetic bottlenecks in inorganic carbon supply [50]. However, while the physiological CA concentration and localization are sufficient to support microalgae growth, the net amount of fixed CO_2_ is insufficient to achieve a significant GHG reduction from high-emission sites. To overcome these problems, exogenous CAs represent a biotechnological strategy to improve inorganic carbon availability in microalgal cultivation systems. However, their practical application remains constrained by several factors, including enzyme selection, delivery mode, high costs, susceptibility to deactivation, and, particularly for free CA, difficulties in recovery and reuse [51]. In addition, CA instability may result not only from protein denaturation, but also from metal ion leaching from the active site [52,53]. To improve enzyme stability and catalytic performance, several approaches have been proposed, including genetic engineering [54,55], chemical modifications [56], and enzyme immobilization [57]. Indeed, several factors must be considered when evaluating the feasibility and scalability of CCU technologies: while the addition of free CA to the culture medium can enhance CO_2_/HCO_3_^−^ interconversion, it limits enzyme recovery and reuse; on the other hand, immobilization can facilitate enzyme recovery and repeated use, but it may compromise catalytic activity depending on the immobilization strategy and support material. In the following paragraphs, the main immobilization strategies employed for CCU applications will be discussed.

## 4. Immobilization Systems for CCU

### 4.1. General Immobilization Methods in CCU

Enzyme immobilization represents a strategic tool to boost catalytic efficiency, enhance enzyme stability under different experimental conditions, such as pH and temperature fluctuations, improve shelf-life, and facilitate enzyme recovery and reusability [58,59,60]. Several immobilization techniques are currently employed, which are mainly classified as “non-covalent methods”, such as adsorption, ionic binding and entrapment/encapsulation, and “covalent methods”, such as covalent binding and cross-linked enzyme [61,62], as schematically illustrated in Figure 2. Independently of the technique used, enzyme immobilization can represent a reliable and efficient approach to reduce extensive downstream processes to make the process cost-effective [62].

Adsorption is a simple and non-invasive enzyme immobilization strategy, based on reversible physical interactions, in which an enzyme is weakly bound to the surface matrix through van der Waals, hydrophobic interactions, ionic and/or hydrogen bonding [63]. These interactions have a minimal impact on protein structure and function, as no harsh chemical treatments are required [64] and their cumulative effect is sufficient to allow a strong interaction between the enzyme and the matrix [65]. Similarly, ionic immobilization can be achieved by using an ion-exchange resin, whose choice depends on the enzyme’s net surface charge, and therefore on the relationship between solution pH and protein isoelectric point (pI) [62,66,67]. Enzymes are predominantly positively charged at pH < pI and negatively charged at pH > pI, determining their interaction with oppositely charged supports. Near the pI, the reduced net charge may weaken the electrostatic contribution to binding, making these systems sensitive to pH fluctuations and potentially favoring enzyme desorption [62]. Changes in ionic strength, temperature, or the presence of competing molecules may also contribute to protein desorption, representing a serious limitation in CCU [47].

Entrapment and encapsulation are based on the physical confinement of enzymes within protective matrices or compartments [68,69]. In entrapment, enzymes are embedded in a polymeric network, such as gels, fibers, or hydrogels, whereas in encapsulation they are enclosed within capsules, particles, or semipermeable membranes [68,70,71]. Since these methods do not require covalent bonding between the enzyme and the support, they generally reduce the risk of chemically induced conformational changes and activity loss. Matrix thickness, pore size, and membrane permeability play key roles in preventing enzyme leakage while allowing the free diffusion of substrates and products [62,70]. Enzymes can be incorporated either during matrix formation or after the network generation.

Covalent immobilization techniques include strategies that differ in the orientation of the enzyme on the support, the number and position of attachment points, and the use of crosslinkers [62,72]. Compared with physical adsorption, covalent attachment generally provides higher storage and operational stability by reducing enzyme desorption and strengthening enzyme–support interactions [59,65,73,74]. Multiple covalent attachments can further increase protein rigidity and limit conformational flexibility, thereby enhancing overall stability [65,75,76,77]. Usually, the binding between protein and matrix is mediated by bifunctional crosslinkers, such as glutaraldehyde, carbodiimides, or silanes [78,79]. However, covalent immobilization often requires longer reaction times, higher production costs, and careful optimization of parameters, such as pH, temperature, crosslinker concentration, and support functionalization. In addition, excessive rigidification, unfavorable enzyme orientation, or the involvement of residues essential for catalysis may negatively affect enzyme activity. Thus, a precise control of immobilization conditions is required to balance stability and catalytic performance.

Finally, cross-linked enzymes (CLEs), cross-linked enzyme crystals (CLECs) and aggregates (CLEAs) represent a class of support-free strategies in which enzymes are covalently bound in their soluble, crystalline or aggregated state, respectively [78]. In CLEs and CLEAs, the free amino groups of surface lysine residues are mainly responsible for cross-linking with glutaraldehyde polymers. CLEs represent the simplest carrier-free immobilization method, although protein purification is required, thus increasing the overall costs. Furthermore, their practical application is limited by poor chemical and mechanical stability and low reproducibility [80]. Conversely, the first step of CLEC preparation is the obtainment of a highly purified protein. After crystallization, the enzyme has a well-ordered structure, so that cross-linkers will primarily affect the surface groups, leaving the active site fully accessible to the substrate. This results in enhanced catalytic and chemical stability, along with a controllable particle size coupled with high productivity. Moreover, CLECs can be easily recovered and reused, even if the crystallization step dramatically increases the overall costs.

To reduce purification-related costs, an alternative procedure was developed, in which a crude protein extract is first precipitated and subsequently cross-linked, leading to CLEA formation. CLEAs show good operational and storage stability, good reusability and, even if they are all derived from crude extracts, they have high catalytic specificity and selectivity. Furthermore, cross-linking confers enhanced rigidity to the tertiary structure, preventing the dissociation of multimeric enzymes and reducing the risk of denaturation. However, since aggregation strictly depends on the specific enzyme, a case-by-case optimization procedure is required.

In spite of their advantages, these carrier-free systems are often constrained by limitations, including enzyme inactivation, conformational alteration of the enzyme, reduced mass transfer, and lower reaction rate [63,81,82]. Therefore, designing an optimal immobilization strategy requires a meticulous selection of the crosslinker, taking into account the enzyme’s functional groups and the specific bonding chemistry [62,83].

### 4.2. Materials for Enzyme Immobilization

Apart from CLEs, CLECs and CLEAs, all other immobilization techniques require solid support to stabilize enzymes and preserve their activity by enhancing their resistance to the surrounding environment. Many matrices, including inorganic or organic materials with distinct physical and chemical properties, have been proposed to optimize protein immobilization, with the common goal of preserving enzyme activity [84]. Choosing an appropriate material is crucial because it strongly influences enzyme catalytic performance. Support suitability depends on key structural factors, such as geometry, size, pore diameter, surface area, and the degree of functionalization [85]. An ideal carrier should be cost-effective, inert, stable, mechanically strong, and capable of enhancing enzyme specificity or activity, while reducing product inhibition and preventing microbial contamination [68]. Inorganic supports offer high mechanical, chemical, and thermal stability, large surface areas, and improved reusability, whereas organic supports are favored for their biocompatibility, mild processing conditions, and sustainability.

Among inorganic supports, silica gels and metal oxides are the most widely used. Silica is extensively used due to its strong thermal and chemical stability and robust mechanical properties, high surface area and porous structure. All these features provide excellent sorption capacity, enabling efficient enzyme attachment while minimizing diffusional limitations [86]. Additionally, the presence of surface hydroxyl groups facilitates functionalization with different crosslinkers [87]. Mesoporous silica, SBA-15, dendritic mesoporous silica and sol–gel silica beads have all been investigated for enzyme immobilization. Recent work on dendritic mesoporous silica highlights how their open radial channels and large pore entrances reduce diffusion limitations [88].

Among metal inorganic oxides, titanium, aluminum and zirconium oxides are well-known for their chemical inertness, high stability, mechanical robustness and good sorption capacity. The presence of hydroxyl groups on their surface renders these materials highly hydrophilic, enhancing surface modification that favors the formation of stable enzyme-matrix interactions. Nanostructured materials provide a large surface area, leading to higher enzyme loading on the matrix surface. Nanoparticles show minimal diffusional limitations, as enzymes are attached to the surface of the nonporous particles, ensuring high catalytic activity retention due to the accessibility of the active site. In particular, iron oxide nanoparticles can combine short diffusion paths with magnetic separation, significantly improving their recovery and reusability [89,90].

Among organic supports, chitosan, alginate and silk-fibroin-based hydrogels are the most widely used [91]. Chitosan is a natural, sustainable, hydrophilic and chemically modifiable polymer, which can be assembled in different forms and shapes. The primary amino groups on the chitosan backbone are responsible for electrostatic interactions and covalent bonding required for enzyme immobilization [92]. However, in biological systems, where pH fluctuations may occur, chitosan can undergo protonation, alter its surface charge and potentially affect crosslinking efficiency. Besides chitosan’s natural binding sites, bifunctional crosslinkers, such as glutaraldehyde and genipin, are often employed to improve mechanical stability, even if they can alter pore structure and mass transfer [92,93]. Due to its high biocompatibility and natural origin, chitosan can be employed in biological systems [94]. Alginate, on the other hand, is generally employed for enzyme entrapment under mild experimental conditions. Its gel network results from the interaction between calcium ions and carboxyl groups of uronic acid residues in alginate chains. The presence of these negatively charged carboxyl groups is responsible for its water solubility [94,95]. The literature provides contrasting evidence on alginate-based enzyme immobilization. While the relatively low mechanical stability of alginate gels and their diffusional limitations can affect substrate and product transport [96], the entrapped enzyme exhibits good catalytic activity, despite enzyme leaching often being observed [94]. Besides these biopolymeric matrices, biochar, obtained through pyrolysis of lignocellulosic biomass or non-lignocellulosic feedstocks, is emerging as a sustainable carbon-based support that combines enzyme immobilization with waste biomass valorization [97,98,99,100,101]. Usually, enzyme immobilization is achieved by adsorption and covalent bonding, strategies that can improve the pH, thermal, storage, and operational stability of the immobilized enzyme. However, biochar is still a pioneering material to be used for enzyme immobilization, as mass transfer, immobilization efficiency, and long-term reusability remain poorly characterized [97].

Recently, novel materials emerged as innovative supports for enzyme immobilization, such as metal–organic frameworks (MOFs), covalent organic frameworks (COFs), and hydrogen-bonded organic frameworks (HOFs). These materials serve as excellent carriers for immobilized enzymes due to their high porosity, surface area, controlled pore structure and designable functionality [102,103,104].

MOFs are a class of supports characterized by modular construction of their components, in which organic links and metal-based nodes are assembled to form extended networks [103,104,105]. This results in precise tailoring, a high degree of crystallinity, remarkable surface areas and pore volumes [106]. When enzymes are encapsulated within MOF structures, the enzyme is effectively protected within a confined catalytic environment [107,108]. Their application is encouraging but still at a primordial stage, mainly due to aqueous instability, pH tolerance, synthesis costs and scaling-up. Moreover, because the framework integrity entirely relies on coordination bonds, metal ion leakage and structural degradation can occur. For these reasons, MOFs are still preferred for specific high-value applications rather than for large-scale supports. Different is the case of COFs, where covalent bonds are responsible for framework stabilization, offering higher stability, uniform pore size distribution and excellent thermal stability. Within these structures, encapsulated enzymes retain their native conformation. However, the size compatibility between pore structure and target enzymes is still a critical parameter to be considered, along with the harsh conditions required for their synthesis [107].

Finally, HOFs are a novel porous crystalline material in which weak interactions, such as hydrogen bonding between organic tectons, are responsible for cage stability. In this case, mild conditions are required to immobilize the enzyme, and the absence of metal confers biocompatibility [107,109]. Different methods and materials are summarized in Table 1, where advantages and limitations are reported. It should be noted that cost-related limitations associated with some immobilization methods are based on qualitative assessments reported in the literature, as the available studies generally lack the standardized technical and economic data required for a robust techno-economic comparison.

### 4.3. Immobilization CAs Applied to CCU

As discussed above, CAs can act as efficient biocatalysts in carbon mitigation processes. Within the CCU framework, the deployment of exogenous CAs represents an innovative strategy to maximize carbon fixation. However, the enzyme, in its free form, cannot be recycled and might be susceptible to degradation and proteolytic events. To overcome these bottlenecks, its successful immobilization is essential, as this could extend operational lifetime and allow enzyme recycling [110,111]. Once immobilized, other factors might be responsible for a successful CCU, such as reactor geometry and transport phenomena. The first strongly influences enzyme performance, being responsible for an efficient aeration system, which in turn is responsible for increasing CO_2_ concentration and local CO_2_ availability. On the other hand, enzymatic reactions are constrained by transport phenomena, in particular by internal diffusion pathways, proton transport, and bicarbonate diffusion, which are directly dictated by the structural morphology and nature of the chosen support. Moreover, during repeated reuse cycles, a progressive loss of enzymatic activity can occur; however, the available studies generally do not clarify whether this is due to enzyme leaching, denaturation, or a combination of both mechanisms.

Different supports have been used to immobilize CAs, as summarized in Table 2 and schematically represented in Figure 3. In the case of silica-based systems, Zhang and colleagues demonstrated that immobilizing bovine CA on nonporous silica nanoparticles (NPs) improved enzyme loading, activity and thermal and chemical stability, with smaller nanoparticles yielding the best performance compared to porous micron-sized supports [112]. In particular, the best ratio between enzyme loading and catalytic activity was observed after 90 min incubation, with a final yield of 45 mgCA/gNP, and a relative activity of 100%. Hydration was measured by a manometric method, using a stirred tank reactor. Similar results were obtained by Fei and colleagues who used an amine-functionalized SBA-15 to covalently immobilize bovine CA [113]. Results indicated that the immobilized protein showed good reusability, as the catalytic activity was retained up to 20 cycles, and the storage stability was similar to that of free CA. Vinoba used a human CA on gold nanoparticles assembled over thiol-functionalized mesoporous SBA-15, demonstrating that silica can retain CA activity while improving reuse and stability [114,115]. The amount of CaCO_3_ precipitated by functionalized particles was similar to that obtained with free CA, and their activity was retained up to 20 days at 25 °C and for up to 20 recycling runs, with a similar hydration ability to that observed for free CA. Hsieh and colleagues successfully entrapped a bacterial CA from *Sulfurihydrogenibium azorense* into biomimetic silica support, with a 100% entrapment efficiency and an activity recovery of 91% compared to free CA [116]. The immobilization strategy enhanced enzyme longevity, as the residual activity after 35 days of storage at 25 °C was two-fold higher than free CA, along with good reusability, as entrapped CA retained 86% activity, up to 10 consecutive cycles. However, when tested for the ability to sequester CO_2_ as CaCO_3_, the immobilized CA showed a slightly longer reaction time compared to the enzyme in its free form. Focusing on sustainability, research moved towards the use of natural polymeric matrices, such as alginate and chitosan, known to be biocompatible. Yadav et al. [117] successfully used alginate-immobilized bovine CA to promote CO_2_ transformation to calcite, and the immobilized enzyme retained about 70% of its initial activity up to six cycles. Oviya et al. [118] reported immobilization of bacterial CA in a chitosan-alginate hydrogel for CO_2_ sequestration. In this case, the optimum temperature was 37 °C with 100% activity for free and immobilized CA. Shen et al. [119] reported CA immobilization on chitosan-coated cotton fibers, in which glutaraldehyde induced enzyme immobilization and CLEAs formation. Immobilization preserved enzyme activity, as about 50% of the initial activity was retained, whereas the free protein reached 2% activity after 30 days of incubation in an accelerated longevity test. The innovative material showed a 66.7% CO_2_ capture efficiency and up to 1-year stability (85%). To improve protein recovery and reusability, magnetic supports were investigated. Magnetic nanoparticles and magnetic CLEAs have been studied for enzymatic CO_2_ capture, showing the practical value of combining catalytic density with magnetic recovery [120,121,122]. As an example, Peirce and colleagues crosslinked bovine CA on magnetic nanoparticles to obtain CLEAs upon precipitation with glutaraldehyde [121]. Even if the binding efficiency and residual activity were successful, the nanoparticles failed to efficiently absorb CO_2_, probably because of the CLEAs distribution at the gas–liquid interface, as well as possible enzyme inactivation during cross-linking. Kim and colleagues developed a 3D-printed platform that serves as an interfacial device to accommodate immobilized bovine CA. The enzyme was immobilized on polystyrene-poly(styrene-co-maleic anhydride) fibers (PS-PSMA) via an enzyme precipitate coating (EPC) protocol. The hydratase activity of EPC-CA/fibers was 1.53 ± 0.06 WAU/mg fiber, 11 times higher than CA COV-CA/fiber (CA covalently immobilized on fibers) used as a control. However, no information on the free enzyme and on immobilization efficiency was reported. Interestingly, the authors calculated the CO_2_ conversion rate of the system, demonstrating that the maximum rate of 78.3 mmol/min/m^2^ was obtained at the aqueous interface. Moreover, the CA-loaded interfacial device demonstrated excellent stability, retaining 99.3% of its initial conversion efficiency after 10 reuse cycles and 88.2% after 459 days of storage in an aqueous buffer at 4 °C [123]. Finally, MOF-based CA systems represent a newer direction, as demonstrated by Jiao and colleagues, who used His-tagged hCA II immobilized on Ni-based MOF nanorods to improve storage, temperature and pH stability [108]. The authors demonstrated that the presence of the tag positively influenced the binding to the nanorods.

The overall applications converge on the same principle: CA immobilization improves process feasibility by enhancing enzyme stability and enabling its reuse, allowing the reduction in process costs up to 50%, and making it a powerful tool for industrial implementation of CO_2_ capture and conversion processes. However, despite these advantages, the overall performance remains strongly dependent on mass-transfer phenomena, which can limit process efficiency [47,124,125,126,127,128].

Although these systems are not based on the direct use of biological systems, they pave the way for biological carbon capture and utilization (bio-CCU) applications. CA-immobilized supports can be employed not merely as particle suspensions, but as modular process units, facilitating their integration with microbial metabolism that utilizes the captured CO_2_ as a carbon source for the production of value-added compounds.

## 5. The Biological Route: Bio-CCU for Carbon Conversion

Biological carbon capture and utilization (bio-CCU) can be defined as the use of living cells or biological catalysts to transform inorganic carbon into biomass, bioproducts or metabolic intermediates. Compared with conventional chemical capture, bio-CCU separates CO_2_ from a gas stream, which is metabolized into molecules with economic value. Depending on the biological source, these molecules may include proteins, lipids, carbohydrates, pigments, extracellular polymers, organic acids, biofuels, or precursors to be used in biorefineries [129].

### 5.1. Microalgae Mediated Bio-CCU

Autotrophic microorganisms utilize sunlight to drive CO_2_ fixation into organic compounds that serve as building blocks for their growth, thus effectively storing carbon in biomass and representing an excellent candidate for bio-CCU [130]. Among them, microalgae and cyanobacteria have gained attention for their ability to convert, via photosynthesis, inorganic carbon into organic carbon-based compounds with economic value [131].

Compared to terrestrial crops, microalgae and cyanobacteria are especially attractive because they possess relatively high photosynthetic efficiency, rapid growth, ability to grow on non-arable land, and capacity to thrive in wastewater streams [132]. Their faster growth rate allows them to fix CO_2_ at a rate up to 50 times higher than that of terrestrial plants [133].

Microalgae-mediated CCU has considerable potential to be a good strategy for enhancing carbon capture, but several limitations still hinder their full exploitation at a large scale. These limitations are mostly rooted in technical factors, with inefficiency in CO_2_ delivery being the most important. Atmospheric CO_2_, the most abundant and sustainable source, has a very low concentration (about 0.04%) and dissolves slowly [134]. In aquatic systems, the chemistry of inorganic carbon is central. Once in water, CO_2_ is hydrated to carbonic acid (H_2_CO_3_), which subsequently dissociates into bicarbonate (HCO_3_^−^), a form of inorganic carbon that can enter the cells. Under equilibrium conditions with atmospheric air, dissolved CO_2_ may represent less than 1% of total dissolved inorganic carbon (Ci), which includes CO_2_, HCO_3_^−^ or CO_3_^2−^, whose ratio is strongly influenced by the pH of the medium. During microalgae growth, high CO_2_ concentration can lower the culture pH and inhibit cell growth, while insufficient CO_2_ transfer can limit productivity [133,135]. As an adaptive response to external inorganic carbon limitation, cyanobacteria and microalgae rely on carbon-concentrating mechanisms (CCMs) to enhance photosynthetic carbon fixation by increasing CO_2_ availability in the Rubisco microenvironment. Microalgae CCMs are summarized in Figure 4.

Unlike higher plants, in which CO_2_ uptake mainly occurs by passive diffusion, cyanobacteria and microalgae CCMs combine CO_2_ diffusion with energy-dependent uptake systems for inorganic carbon (iC), especially HCO_3_^−^ [48]. The induction of the CCM is strongly linked to light availability and low-Ci conditions, since light provides the energy required for iC uptake while regulating the expression, localization, and assembly of several CCM components. A central feature of this mechanism is the rapid equilibration between CO_2_ and HCO_3_^−^, a process mediated by CAs, which plays a fundamental role in the CCM of both microalgae and cyanobacteria. CAs cooperate with Rubisco, thereby increasing the local CO_2_ concentration around Rubisco and improving carboxylation efficiency. Once internalized, HCO_3_^−^ can be converted back into CO_2_ by CAs, facilitating CO_2_ delivery to Rubisco-containing compartments [33,34]. Then, Rubisco catalyzes the carboxylation of ribulose-1,5-bisphosphate, RuBP, producing two molecules of 3-phosphoglycerate. This Calvin–Benson cycle intermediate can then enter central carbon metabolism and contribute to the formation of acetyl-CoA, a key precursor for the biosynthesis of lipids and other value-added compounds [28,35]. Although cyanobacteria and eukaryotic microalgae both rely on CCM to enhance photosynthetic CO_2_ fixation, their structural organization differs substantially. In cyanobacteria, CCM mainly relies on active HCO_3_^−^ uptake and CO_2_ uptake systems coupled to carboxysomes, proteinaceous microcompartments in which RuBisCO and CAs are co-localized. Within carboxysomes, CAs convert accumulated HCO_3_^−^ into CO_2_ in close proximity to RuBisCO, thereby increasing the local CO_2_ concentration and improving carboxylation efficiency. In eukaryotic microalgae, CCMs are generally more compartmentalized, involving inorganic carbon transport across the plasma membrane, the chloroplast envelope and, in some species, the thylakoid membranes (Figure 4). Accordingly, CA isoforms can be localized in periplasmic regions, the cytosol, mitochondria, chloroplasts or pyrenoid-associated compartments, where they regulate CO_2_/HCO_3_^−^ interconversion and facilitate inorganic carbon delivery to the photosynthetic machinery. From a bio-CCU perspective, this structural distinction is crucial, as cyanobacteria and eukaryotic microalgae may respond differently to CO_2_ supply, bicarbonate availability and pH changes, depending on how their CCMs and CA isoforms are organized. Therefore, carbon uptake should not be considered only as a function of CO_2_ concentration in the inlet gas, but also as the result of inorganic carbon conversion, transport, and local availability both at the cell surface and in proximity to the RuBisCO carboxylation site. CAs are fundamental enzymes in CCM regulation, and for this reason they are sensitive to environmental conditions. Increasing CO_2_ concentration above a certain threshold (>20%) can decrease CA expression, thereby reducing CO_2_/HCO_3_^−^ interconversion [30]. In microalgae-mediated bio-CCU, biological processes and transport phenomena are strongly interconnected, since light distribution, gas–liquid transfer, carbonate chemistry, culture pH, nutrient availability, cell density, microbial interactions and bioreactor geometry all simultaneously influence the final CO_2_ fixation rate [30,136]. Optimizing these cultivation parameters represents therefore a first strategy to maximize carbon-fixing potential and enhance carbon capture [36]. However, even under optimized cultivation conditions, each strain possesses its own metabolic features, which ultimately determine its maximum carbon fixation capacity. Some examples of microalgal strains commonly used for CO_2_ capture are summarized in Table 3.

### 5.2. Immobilized CAs Applied to Bio-CCU

An integrated bio-CCU approach can be achieved by combining microalgae with immobilized CAs. In this system, the immobilized enzyme acts as an auxiliary tool by catalyzing the interconversion between CO_2_ and bicarbonate, thereby increasing the availability of inorganic carbon in the microalgal culture medium and facilitating its uptake by the cells. The subsequent biological fixation and utilization of inorganic carbon are instead performed by microalgae through photosynthesis.

To date, only a few studies have investigated the use of immobilized CAs on reusable matrices to support microalgae growth, thus contributing to bio-CCU [148,149,150,151]. Among these, Kim’s group used immobilized bovine CA on composites of carboxylated polyaniline nanofibers to improve *Dunaliella tertiolecta* (ATCC 30929) growth [110]. The authors crosslinked CA to nanofibers and then to magnetic nanoparticles. While both esterase and hydration catalytic activities were successfully evaluated, the authors could not determine the immobilization yield or specific activity of the enzyme.

Accordingly, no data on the amount of protein per nanoparticle was reported. When these nanoparticles were tested on *D. tertiolecta* in the presence of atmospheric CO_2_, the system induced a 1.8-fold increase in microalgae growth after 6 days of incubation, and the immobilized enzyme was able to sustain this growth up to two cycles. Martin’s group worked on a different support to covalently entrap CA to be tested on *Nannochloropsis salina* cultivation [111]. The authors cross-linked CA with glutaraldehyde before encapsulating it into buoyant calcium alginate beads, with an immobilization yield of 80%, but with no indication of the amount of immobilized CA per bead. Notably, as these beads are buoyant, they could theoretically eliminate the need for air sparging to supply CO_2_, as beads may capture CO_2_ directly from the water-air interface. When CA-GA beads were added to microalgae culture in the absence of air sparging, the authors observed a 40% increase in biomass accumulation after 7 days of incubation. However, CA-GA beads were not able to induce biomass growth to the same extent as cells grown in the presence of air sparging. Recently, Wang and colleagues used CA coated onto a polysulfone membrane through a layer-by-layer method [152]. Specifically, they evaluated CA immobilization on membranes consisting of 1-, 3-, and 5-layers, with the 3-layer configuration exhibiting the highest enzymatic activity. However, the authors did not determine the specific catalytic activity or the immobilization yield. When tested on microalgae, the immobilized CA increased biomass growth by approximately 50% after 7 days of cultivation compared to the control. Interestingly, this system also increased the carbon fixation rate by 50% compared with cultures supplied with a commercial air-stone sparger. Furthermore, the membranes could be reused for up to five consecutive cultivation cycles while fully maintaining their growth-promoting effect.

He et al. cultivated microalgae cells within a silk/alginate hydrogel, in which CA was encapsulated in silk nanoparticles and co-embedded with microalgae in the hydrogel [53]. After 7 days of incubation, a 50% increase in biomass accumulation was observed in cells grown in the presence of CA-loaded hydrogel, along with a 50% enhancement in carbon fixation rate, with respect to control cells. The hydrogel was further improved by a microfluidic system, which allowed moisturization of the gel. Relative activity of the encapsulated CA was about 85%; however, no data on protein immobilization yield was reported.

Thakur et al. immobilized CA from *Corynebacterium flavescens* onto ZIF-8/graphene oxide nanocomposites through covalent bonding and onto biochar through physical adsorption [153]. Although both systems achieved high immobilization yields, biochar was found to better preserve the catalytic activity, maintaining it at a level comparable to that of the free enzyme. Accordingly, when tested in a *Chlorella* sp. culture, only the biochar-immobilized CA resulted in an 80% increase in microalgal growth. However, no information was provided regarding the CO_2_ fixation rate or the reusability of the immobilized enzyme. Liu and colleagues encapsulated a recombinant dimeric CA from *Sulfurihydrogenibium azorense* into silica particles, which were then dispersed in epoxy resin [154]. The enzyme loading directly increased with the amount of silica particles added, reaching the best results when 200 mg of silica particles were used for the preparation of silica coatings, as the highest specific activity was reached. The innovative material showed a 30% increase in *Chlorella* biomass concentration after 5 days of growth. Wang and colleagues used delignified bamboo to obtain cellulose as a structural building block for particle formation [155]. Bovine CA was covalently encapsulated through a Schiff-base link to obtain 6 mm particles. When tested for their catalytic activity, they showed lower activity with respect to free CA, and no data on encapsulation efficiency was reported. Finally, CA-loaded particles were able to induce *Chlorella* growth by 16% after 8 days of incubation; however, it was not possible to recover CA-loaded particles after use. The main outcomes of the studies described above are summarized in Table 4.

Overall, the comparison among these studies highlights several limitations that currently hinder a comprehensive assessment of the potential of immobilized CAs for microalgae-based bio-CCU. First, enzyme loading efficiency, immobilization yield, and the specific catalytic activity of the immobilized enzyme are not always reported, limiting a direct comparison of immobilization performance.

Moreover, although several studies have demonstrated the possibility of recycling immobilized CA, reusability is often evaluated through consecutive cycles performed in clean buffer systems rather than under actual microalgal cultivation conditions. This distinction is particularly relevant because prolonged operation in biological systems may introduce additional factors, including biofouling, cell adhesion or entrapment, accumulation of extracellular biomolecules, changes in pH and ionic strength, and progressive enzyme leaching and/or denaturation, all of which may negatively affect the catalytic performance of the immobilized system.

The studies reported to date are therefore very promising but should still be regarded as pioneering proof-of-concept investigations, as they have predominantly been performed at a laboratory scale and under conditions that do not fully reproduce industrial CO_2_ streams. In particular, most experiments have been conducted using atmospheric CO_2_ or simplified CO_2_-enriched conditions, whereas industrial flue gases can contain substantially higher CO_2_ concentrations together with other gases, which may affect both CA stability and microalgae physiology. The tolerance of immobilized CAs and their supporting matrices to these potentially inhibitory compounds represents an important knowledge gap for future studies.

## 6. Conclusions

Carbon Capture and Utilization allow the transformation of captured CO_2_ into high-value products, fostering the transition toward a circular economy. The integration of microalgae culture medium with exogenous immobilized carbonic anhydrases represents a promising bio-CCU strategy, even if a significant gap between literature claims and industrial feasibility remains. Current literature focuses on critical deployment barriers, including high enzyme production and immobilization costs, low operational biocatalyst stability, mass transfer bottlenecks, energy-intensive harvesting, and photobioreactor design constraints. Critically, delivering sufficient CO_2_ without driving the culture pH to growth-inhibiting levels remains an unresolved issue. Moreover, studies frequently report short-term biomass growth, rather than carbon fixation rates, carbon partitioning or downstream product yields. The analysis of the immobilized CA systems (Table 4) further highlights the limited standardization of available data. Indeed, immobilization yield was not reported in 50% of the studies, reusability was not evaluated in 63% of the cases, while specific CO_2_ fixation rate was not determined in 75% of analyzed papers. This lack of key parameters limits the quantitative comparison of immobilization strategies and the assessment of relationships between enzyme loading, retained catalytic activity, and microalgae performance. Future research should adopt a multidisciplinary approach, focusing on advanced nanostructured supports, hybrid organic-inorganic matrices, and porous functional materials capable of enhancing enzyme performance under harsh operational conditions. More systematic and standardized reports of immobilization yield, enzyme loading, and specific activity will be essential to allow a reliable comparison among immobilized CA systems. These data are necessary to perform techno-economic analyses and life-cycle assessments needed to evaluate the scalability, economic and environmental feasibility of the process at an industrial scale. Overcoming these issues is crucial to establish an integrated microalgae-CA system as a viable industrial technology and to avoid bio-CCU remaining a mere concept.

## Figures and Tables

**Figure 1:**
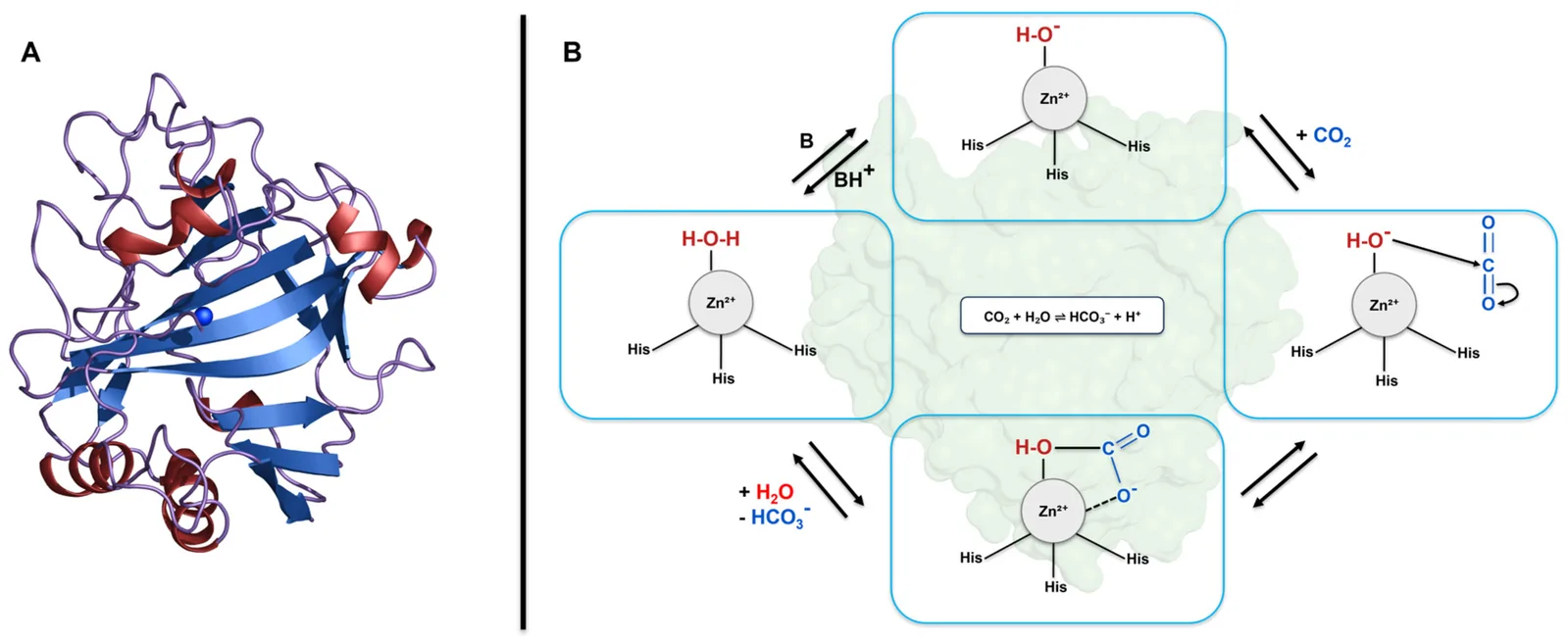
Human carbonic anhydrase II (hCA II) structure and catalytic mechanism schematically represented. (**A**) hCA II (PDB ID: 1CA2) is shown as a representative α-carbonic anhydrase structure. α-Helices are colored in red, β-sheets in blue, and loops in purple, while the catalytic Zn^2+^ ion is highlighted as a bright blue sphere within the active site. (**B**) Schematic representation of the hCA II catalytic mechanism: the Zn^2+^ ion cofactor is coordinated by three amino acid residues from the protein backbone; the fourth metal ion ligand is a water molecule/hydroxide ion that acts as the nucleophile in the enzyme catalytic cycle.

**Figure 2:**
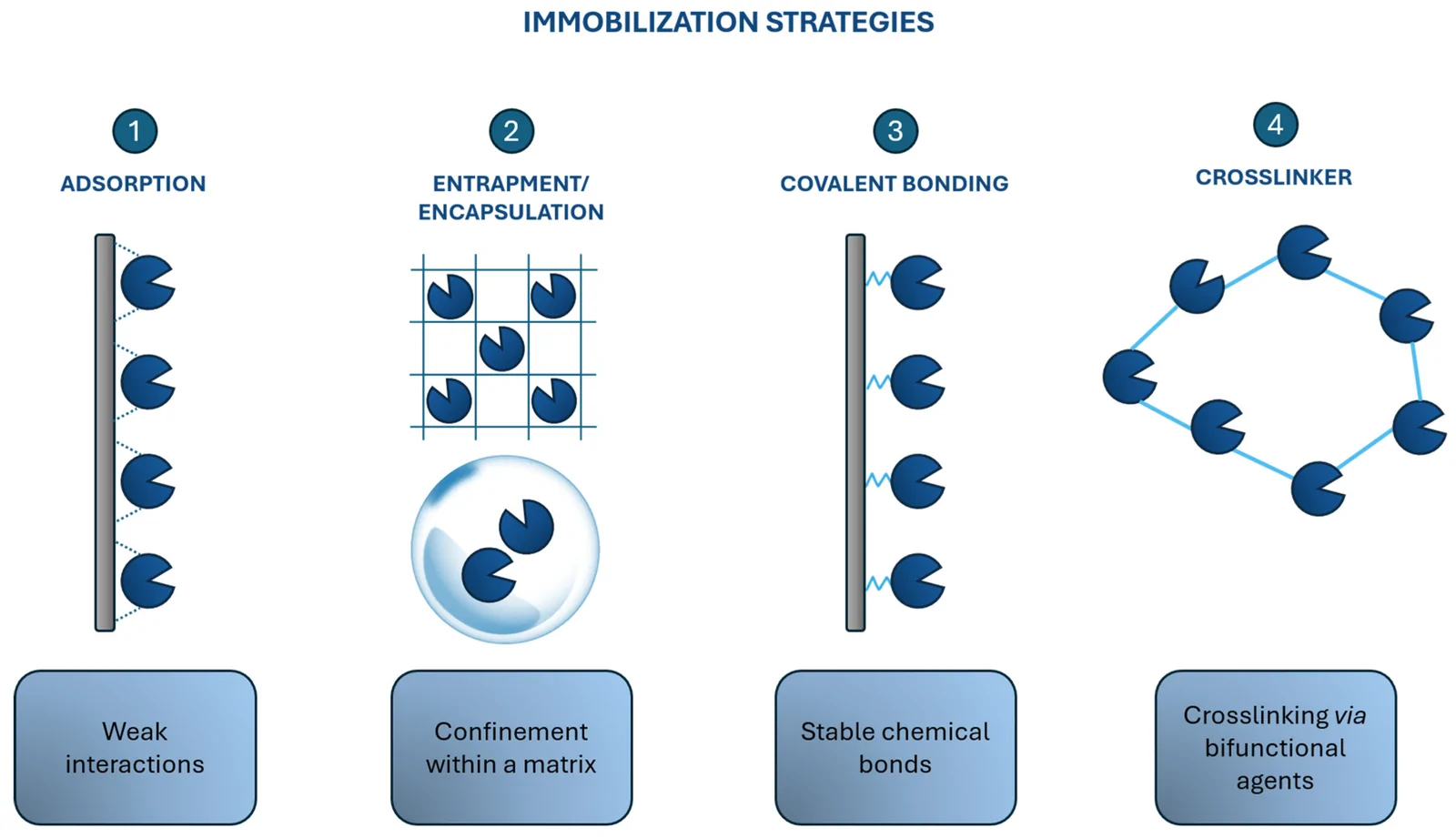
Schematic classification of enzyme immobilization strategies.

**Figure 3:**
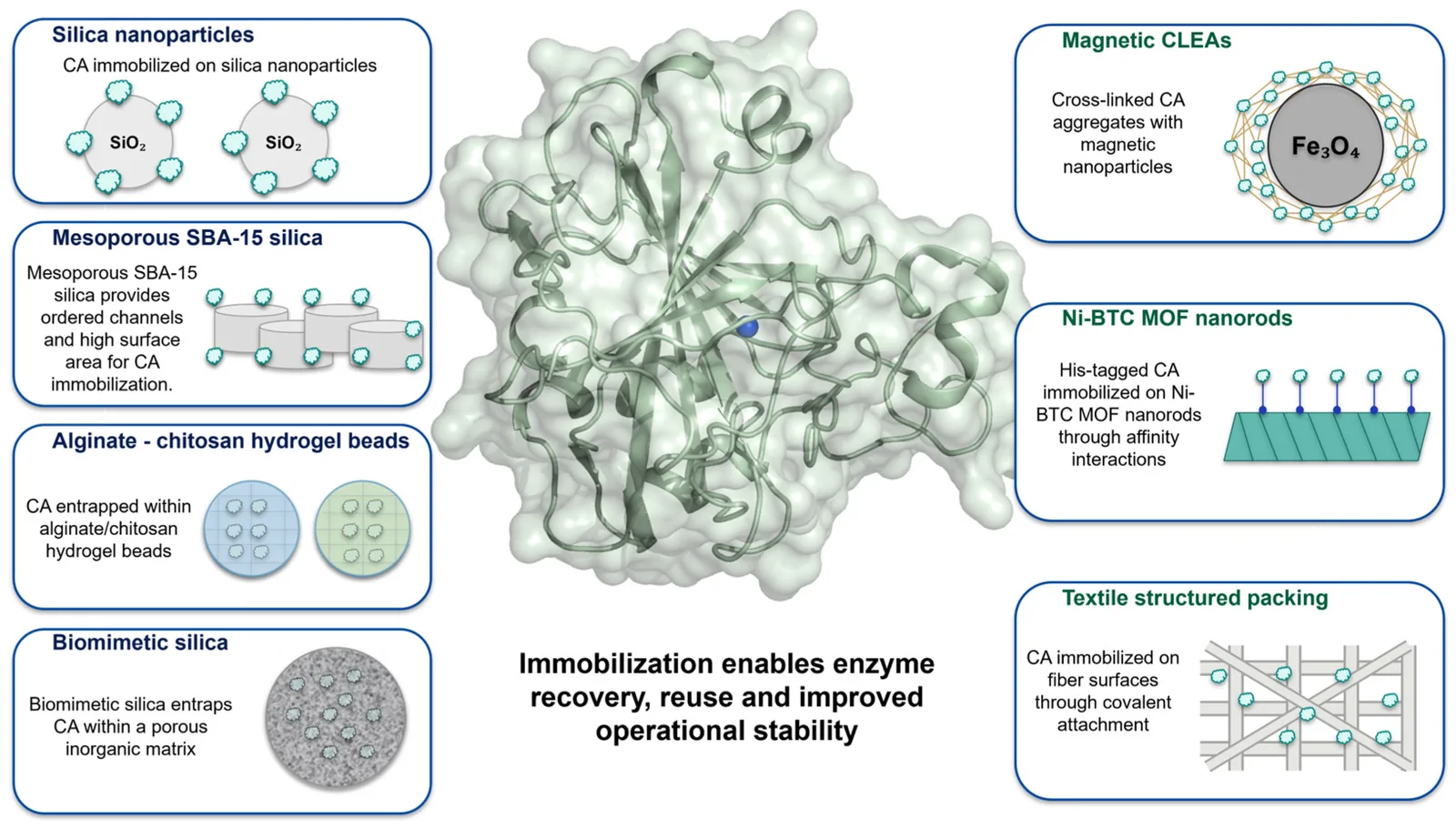
Carbonic anhydrase immobilization platforms for CCU applications. Human carbonic anhydrase II (hCA II, PDB ID: 1CA2) is shown as a representative CA model. The surrounding panels graphically summarize the main CA immobilization supports as reported in Table 2.

**Figure 4:**
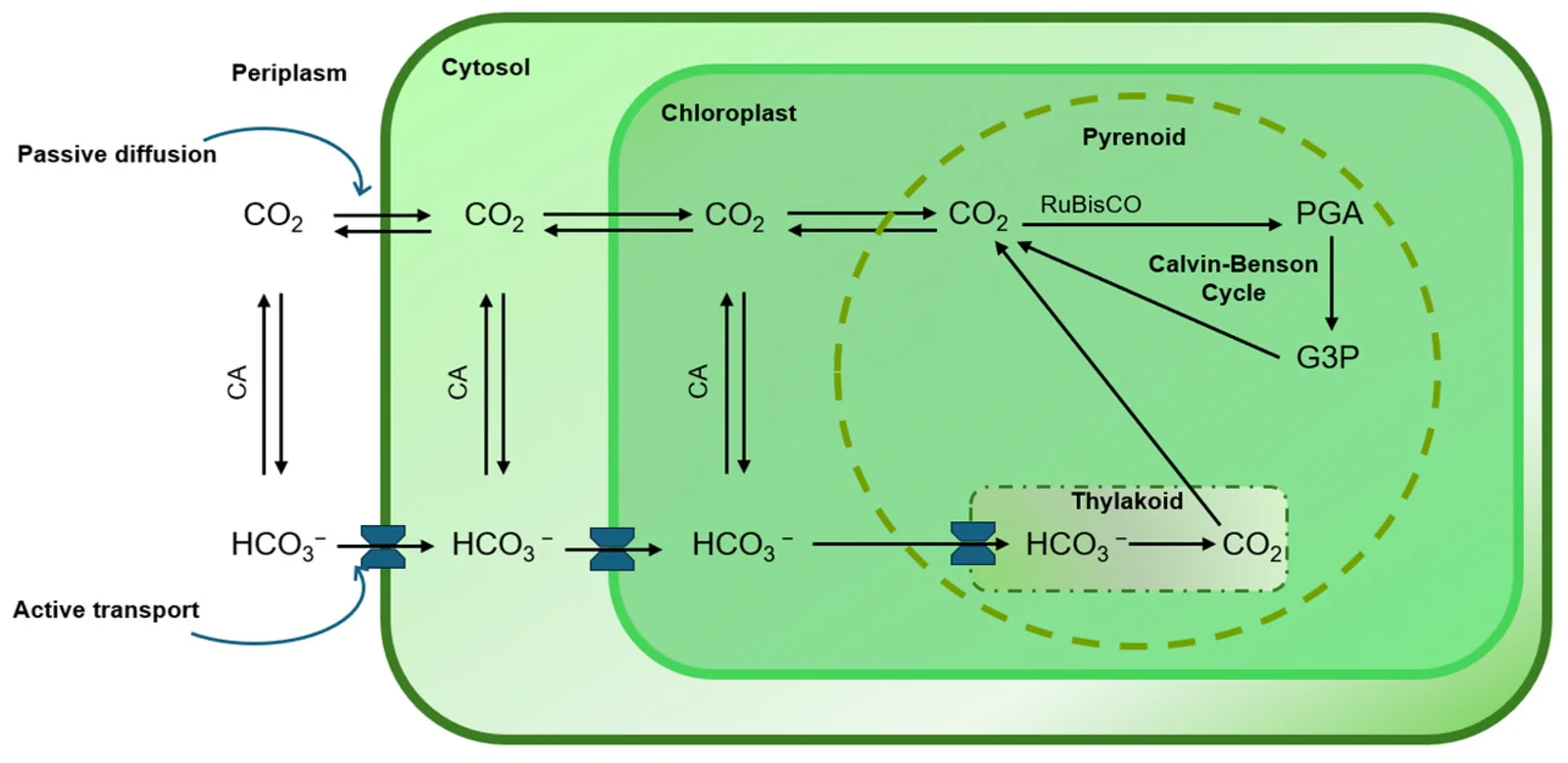
Schematic representation of the Carbon-Concentrating Mechanism (CCM) and carbon fixation pathway in microalgae. PGA, 3-phosphoglycerate; G3P, glyceraldehyde-3-phosphate.

**Table 1:** Overview of non-covalent and covalent enzyme immobilization methods, including materials, crosslinker requirements, enzyme-matrix interactions, main advantages, and operational limitations.

Method	Materials	Crosslinker Requirement	Enzyme-Matrix Interaction	Advantages	Limitations
Adsorption	Silica, polymeric resins, chitosan, cellulose, zeolites, biochar, MOFs, HOFs, COFs	Generally not required	Weak and reversible interactions including Van der Waals forces, hydrophobic interactions, hydrogen bonds, electrostatic interactions)	Simple, inexpensive, mild chemical conditions, good preservation of enzyme structure and activity	Enzyme desorption, sensitivity to fluctuation in pH, temperature, ionic strength, competition with other molecules
Ionic binding	Cellulose, ionic-exchange resin	Generally not required	Ionic interaction between charged amino acid residues and oppositely charged matrix groups	Fast and reversible immobilization, minimal impact on enzyme catalytic activity	Sensitivity to pH and ionic strength fluctuations
Entrapment	Sol–gel silica, alginate, carrageenan, biochar, MOFs, HOFs, COFs	Generally not required; optional for matrix stabilization	Physical confinement within pores or gel/polymer network	Mild conditions, good enzyme preservation	Diffusion limitations, enzyme leakage if pore size is unsuitable
Encapsulation	Liposomes, chitosan/alginate microcapsules, biochar, MOFs, HOFs, COFs	Sometimes required	Physical confinement of the enzyme within semipermeable membranes (no direct interactions)	Good preservation of enzyme structure and activity	Mass transfer limitations; complex preparation; high cost
Covalent immobilization	Activated agarose, epoxy resins, functionalized silica, chitosan, magnetic nanoparticles, biochar	Generally required, e.g., glutaraldehyde, carbodiimides, genipin	Covalent bond between functional groups on the enzyme surface, such as amino, carboxyl, thiol, or hydroxyl groups and activated groups on the support	High chemical stability; low enzyme leakage; good recovery and reusability	Possible enzyme conformational changes; loss of catalytic activity; complex procedures, higher costs
Cross-Linked Enzymes (CLEs)	No solid support required; enzyme aggregates	Yes, commonly glutaraldehyde	Covalent cross-linking mainly between lysine and ε-NH_2_ residues of enzymes and glutaraldehyde	Simple and carrier-free method; increased thermal stability	Poor mechanical stability; possible loss of activity; limited reproducibility
Cross-Linked Enzyme Aggregates (CLEAs)	No solid support required; enzyme aggregates	Yes, commonly glutaraldehyde	Covalent cross-linking mainly between lysine ε-NH_2_ residues of enzymes and glutaraldehyde	High thermal, solvent, and proteolytic stability, low enzyme-purity requirement, high catalyst density, easy recovery	Full recovery is hard to achieve; mass transfer limitations for high molecular weight substrates; possible activity loss; limited reproducibility
Cross-Linked Enzyme Crystals (CLECs)	No solid support required; enzyme crystals	Yes, commonly glutaraldehyde	Covalent cross-linking mainly between lysine ε-NH_2_ residues of enzymes within enzyme crystals and glutaraldehyde, forming a rigid and insoluble 3D network	High operational stability; tunable particle size; high productivity; low enzyme leakage; easy recovery and reusability	Mandatory enzyme crystallization; requirement of highly pure enzyme; difficult procedure; higher costs
Metal–Organic Frameworks (MOFs)-based covalent immobilization	Functionalized MOFs, e.g., amino- or carboxyl-functionalized MOFs	Sometimes required, e.g., carbodiimides/EDC-NHS or glutaraldehyde, depending on functional groups	Covalent interactions between enzyme surface groups, such as amino or carboxyl groups, and activated functional groups on the MOF surface, forming mainly amide bonds	Mild synthetic conditions; simple loading process; high surface area; tunable pore size; retention of catalytic activity; good enzyme accessibility; reusability	Synthesis complexity; recovery and scalability issues; higher costs

**Table 2:** Immobilization strategies, catalytic performance, stability, and reusability of immobilized CAs.

CA Origin	Immobilization Support	Immobilization Method	Immobilization Efficiency	Enzyme Activity		Stability	Reusability	Reference
				Hydration Activity	Esterase Activity			
Human CA	Thiol functionalized SBA-15/mesoporous silica	Adsorption or covalent immobilization	289.13 mg_CA_/g_silica_	n.r.	27.75 mM (immobilized), 13.07 mM (free)	100% residual activity after 20 days at 25 °C	20 cycles	[114,115]
Bovine CA	Nonporous silica nanoparticles	Covalent immobilization	45 mg_CA_/g_NPs_	Relative activity: 100%	n.r.	38% residual activity after 30 days, pH 10.5, 50 °C	n.r.	[112]
Bovine CA	Alginate	Entrapment	7%	3.30 U/bead (immobilized), 3944 U/mg_protein_ (specific activity free)	n.r.	Stable up to 20 days at 4 °C	67% residual activity after 6 cycles	[117]
*B. subtilis* CA	Chitosan-alginate hydrogel	Entrapment	n.r.	163 U/mL (immobilized), free n.r. 480 mg CaCO_3_/mg_protein_ (immobilized), 340 mg CaCO_3_/mg_protein_ (free)	n.r.	93% residual activity after 50 days at 4 °C	n.r.	[118]
Bovine CA	Epoxy-functionalized SBA-15	Covalent immobilization	222 mg_CA_/g_support_	n.r.	3.1 mM (immobilized), 2.4 mM (free CA)	91% residual activity up to 30 days at 4 °C	Up to 20 cycles	[113]
Bovine CA	CLEA and magnetic CLEA	Cross-linking/magnetic cross-linked enzyme aggregate	84%	12.7 WAU/mL (immobilized), 36.2 WAU/mL (free)	n.r.	*E* > 1 for CLEAs compared to *E* = 3.4 for free CA	5 cycles of CO_2_ absorption tests	[121]
Human CA	Ni-BTC MOF nanorods	Coordination/affinity immobilization	31.8%	8296.3 U/mg (immobilized),1.96 U/mg (free)	n.r.	n.r.	65% residual activity after 6 cycles	[108]
*S. azorense* CA	Biomimetic silica	Entrapment/biosilicification	100%	16,887 WAU/mg corresponding to 91% free CA activity	n.r.	62% residual activity up to 35 days at 25 °C	86% residual activity after 10 cycles	[116]
Thermostable bacterial CA	Textile structured packing	Covalent	0.188 U/g_cellulose_	n.r.	n.r.	85% residual activity up to 1 year	10 cycles	[119]
Bovine	PS-PSMA fibers	Covalent attachment and enzyme precipitation coating	n.r.	1.53 WAU/mg fibers (immobilized); free enzyme n.r.	n.r.	88.2% CO_2_ conversion rate up to 459 days at 4 °C	99.3% CO_2_ conversion rate after 10 cycles	[123]

n.r. = not reported; WAU = Wilbur-Anderson Units; *E* = CO_2_ absorption rate enhancement factor.

**Table 3:** Microalgae-mediated bio-CCU: CO_2_ removal efficiency and fixation rate of selected microalgal strains.

Microalgal Species	Biomass Concentration (g L^−1^)	CO_2_ Concentration (vol%)	CO_2_ Removal Efficiency (%)	CO_2_ Fixation Rate (g L^−1^ d^−1^)	References
*Chlorella kessleri*	n.r.	23	n.r.	0.187	[137]
*Desmodesmus* sp. SZ-1	2.7	10	n.r.	0.5	[138]
*Chlorella vulgaris*	n.r.	10–20	40	0.51	[139]
*Chlorella* sp.	2.4	5	5	0.35	[140]
*Scenedesmus* sp.	1.95	10	6.6	2.18	[141]
*Scenedesmus* *dimorphus*	0.58	10	94.6	0.20	[142]
*Scenedesmus* *dimorphus*	1.16	0.235	14.4	0.432	[143]
*Dunaliella salina*	0.25	6	n.r.	0.067	[144]
*Spirulina LAMB 220*	2	15	n.r.	0.4	[145]
*Chlamydomonas* sp.	n.r.	23	n.r.	0.18	[137]
*Chlorococcum* *humicola*	0.896	5	n.r.	0.154	[146]
*Chlorella vulgaris*	1.3	5	n.r.	0.214	[146]
*Spirulina* sp. LEB18	1.98	n.r.	80.5	0.231	[147]

n.r. not reported.

**Table 4:** Immobilized CA systems used to enhance microalgal growth and CO_2_ utilization, including support materials, immobilization methods, enzymatic activity, immobilization yield, and reusability.

CA Source	Immobilization Support	Immobilization Method	Microalgae Strain	CA Activity Assay	Immobilization Yield	Growth Enhancement	CO_2_ Capture Enhancement	Reuse on Microalgae (Cycles)	Reference
Bovine	Nanofibers	Crosslinking	*D. tertiolecta*	p-NPA and W.A.; specific CA activity not determined	n.r.	1.8-fold increase	n.r.	2	[110]
Bovine	Alginate	Crosslinking	*N. salina*	p-NPA and W.A.; specific CA activity not determined	80%	40% increase with respect to algae not supplied with CO_2_	n.r.	3	[111]
n.r.	Polysulfone membrane	Adsorption	*C. vulgaris*	W.A.; specific CA activity not determined	n.r.	50% increase	50% increase	5	[152]
Bovine	Silk fibroin nanoparticles co-embedded with microalgae in a sodium alginate hydrogel	Encapsulation	*Chlorella* sp.	p-NPA; specific CA activity not determined	n.r.	50% increase	50% increase	n.r.	[53]
*C.* *flavescens*	Graphene oxide nanocomposites	Covalent	*Chlorella* sp. (TKGA8)	p-NPA; 535.67 U/mg immobilized CA; 597.30 U/mg free CA	94.5%	80% increase	n.r.	n.r.	[153]
*C.* *flavescens*	Biochar	Adsorption	*Chlorella* sp. (TKGA8)	p-NPA; 473.86 U/mg 597.30 U/mg free CA	82%	No effect	n.r.	n.r.	[153]
*S. azorense*	Silica particles dispersed in epoxy resin	Encapsulation	*C. vulgaris*	0.176 WAU/ cm^2^	0.12 mg/cm^2^	29% increase	n.r.	n.r.	[154]
Bovine	Cellulose	Covalent	*C. vulgaris*	2.2 WAU; 2.9 WAU free CA	n.r.	16% increase	n.r.	n.r.	[155]

n.r. = not reported; p-NPA = p-nitrophenyl acetate assay; W.A. = Wilbur-Anderson assay.

## Data Availability

No new data were created or analyzed in this study. Data sharing is not applicable to this article.

## Abbreviations

The following abbreviations are used in this manuscript:

GHGs	Greenhouse gases
CCU	Carbon capture and utilization
CCS	Carbon capture and storage
CCUS	Carbon capture utilization and storage
bio-CCU	Biological carbon capture and utilization
CAs	Carbonic anhydrases
Gt	Gigatons
EU	European Union
USD	United States dollars
CAGR	Compound Annual Growth Rate
hCAII	Human carbonic anhydrase II
CLEs	cross-linked enzymes
CLECs	cross-linked enzyme crystals
CLEAs	cross-linked enzyme aggregates
MOFs	Metal–organic frameworks
COFs	Covalent-organic frameworks
HOFs	Hydrogen-bonded organic frameworks
NPA	p-nitrophenyl acetate assay
W.A.	Wilbur Anderson assay
W.A.U.	Wilbur Anderson unit
SBA-15	Santa Barbara amorphous-15
